# Composition-Dependent Microwave Shielding Performance of Kaolinite/Chopped-Strand Composites for Wide Frequency Range

**DOI:** 10.3390/nano16191267

**Published:** 2026-10-08

**Authors:** Mehriban Emek

**Affiliations:** Faculty of Aviation and Space Sciences, Department of Aviation Management, Adana Alparslan Türkeş Science and Technology University, Adana 01250, Turkey; emekmehriban@gmail.com

**Keywords:** microwave shielding effect, kaolinite, mixed-oxide, chopped strands, matrix composites

## Abstract

The increasing demand for high-frequency electronic and communication systems has intensified the need for lightweight and efficient electromagnetic interference (EMI) shielding materials. In this study, the conventional oxide mixing method was used to form kaolinite-chopped strand composites. Natural kaolinite powder was calcined at 450 °C for 4 h to remove volatile and organic components before composite fabrication. Kaolinite/chopped-strand epoxy composites were fabricated and evaluated for their microwave shielding effectiveness in the 0–4.4 GHz frequency range. Composites with different kaolinite-to-chopped-strand weight ratios were prepared using a controlled mixing and molding process to obtain thin specimens with a uniform thickness of 1.4 mm. X-ray diffraction, scanning electron microscopy, and energy-dispersive X-ray spectroscopy were used primarily to characterize the kaolinite powder. Because composite-level mapping was not performed, the uniformity of filler dispersion is interpreted cautiously. Microwave shielding effectiveness was measured using a two-port vector network analyzer (VNA). The composite containing 80 wt.% kaolinite and 20 wt.% chopped strands showed the highest shielding performance, with a maximum total shielding effectiveness of 21.57 dB at 0.58 GHz. The observed shielding behavior is consistent with absorption-dominated attenuation supported by S-parameter-derived reflection, transmission, and absorption trends. This can arise from polarization losses and interfacial charge accumulation in heterogeneous dielectric composites. The results indicate that kaolinite/chopped-strand epoxy composites are low cost and sustainable materials for broadband microwave shielding applications.

## 1. Introduction

Rapid advancement in high-frequency electronic systems, wireless communication technologies, radar platforms, and sensing devices has led to a significant increase in electromagnetic radiation in the GHz frequency range. As a consequence, electromagnetic interference (EMI) has become a major technological and environmental issue, adversely affecting the performance, reliability, and durability of electronic equipment, and potentially endangering human health [1,2,3,4,5,6]. Hence, the development of effective electromagnetic shielding materials to suppress unwanted electromagnetic waves has been a major research priority.

A combination of reflection, absorption, and multiple internal reflections of incident electromagnetic waves is usually employed to realize electromagnetic interference (EMI) shielding [7,8,9]. Of these, absorption-dominated shielding is particularly attractive because it reduces secondary electromagnetic pollution from reflected waves. The shielding effectiveness of a material depends on several inter-related factors such as electrical conductivity, dielectric and magnetic losses, impedance matching, microstructure, filler dispersion, and interfacial polarization [10,11,12]. Therefore, an ideal EMI shielding material should have low density, thin thickness, high shielding effectiveness over a wide frequency range, good mechanical stability, corrosion resistance, and be cost effective [13].

To fulfill such requirements, many materials have been studied for EMI shielding and microwave absorption, such as carbon fibers [14], magnetic metal powders [15], conducting polymers, ferrites, graphene-based materials [16,17], MXenes, metal–organic frameworks, and hybrid carbon-based composites [18]. Moreover, recent research has focused on multifunctional materials combining electromagnetic shielding with antibacterial activity or mechanical flexibility for wearable and eco-friendly applications [19]. However, with the increasing operation at higher frequencies and wider bandwidth requirements of modern communication and radar systems, many conventional shielding materials and frequency-selective structures are no longer adequate for effective broadband attenuation [20,21,22]. Such limitation has fueled the interest in the development of new composite materials with tunable electromagnetic properties.

Radar-absorbing and stealth-related materials are particularly sensitive to the reduction in both transmission and reflection of incident electromagnetic waves [23,24]. In these systems, effective absorption of microwave energy in the material is key to suppressing radar detectability. Porous and heterogeneous composite structures are particularly advantageous in this respect, providing multiple scattering centers, enhanced interfacial polarization, and improved impedance matching, which lead to increased attenuation of electromagnetic wave.

Kaolinite is a natural clay mineral with the chemical formula of Al_2_Si_2_O_5_(OH)_4_. There is significant interest to explore kaolinite due to its layered aluminosilicate framework, surface reactivity, eco friendliness, as well as low price [25]. Owing to its effective adsorption ability, remarkable physical strength, and chemical stability, it is commonly applied in ceramics, paper industries, refractories, drilling fluids, mineral processing, and environmentally friendly usages [26,27,28,29,30,31]. The two-dimensional hydrogen-bonded aluminosilicate layers structure which exhibits a lamellar nature can bring the benefits of electromagnetic interference shield based on interfacial polarization and a dielectric losses mechanism [32,33]. Despite these advantageous properties, kaolinite has been much less studied as a functional filler for microwave shielding applications compared to conductive or magnetic materials.

Chopped glass fiber strands, especially E-glass fibers, are widely used as reinforcing materials in polymer composites because of their high strength-to-weight ratio, corrosion resistance, electrical insulation, and low cost [34,35]. The chopped strands may influence the electromagnetic response through changes in interfacial polarization, scattering, impedance matching, and composite morphology. However, their individual contribution cannot be isolated without measurements of epoxy/chopped-strand control specimens [35,36]. The aspect ratio, dispersion, and concentration of chopped strands may influence the electromagnetic response of the composite. However, in the present study, the final fiber-length and aspect-ratio distributions after mechanical crushing and ball milling were not measured.

The reported shielding effectiveness values for the different composite systems, e.g., Wollastonite/PANI/Colemanite [37], carbon fiber/epoxy composites [38], BaFe_12_O_19_ nanoparticles [39], graphene-based nanocomposites [40], and kaolinite-based polymer composites [41] working in lower frequency ranges, are promising.

The shielding efficiency defines the ability of the material to attenuate the incoming electromagnetic waves. A transmission coefficient of 10 dB corresponds to a transmitted power of 10%. Using the positive shielding-effectiveness convention, this corresponds to a total shielding effectiveness of 10 dB, indicating 90% attenuation of the incident electromagnetic power [42,43].

However, the reported kaolinite containing systems are limited by either narrow operation bandwidths or relatively low shielding effectiveness or thicker sample requirements. Furthermore, systematic studies on thin kaolinite/chopped-strand epoxy composites in a broad frequency range (0–4.4 GHz) are still rare.

However, there is a clear lack of research on the combined use of kaolinite and chopped strands in thin epoxy-based composites for broadband microwave shielding applications, although they have interesting properties. In particular, the effect of the ratio of kaolinite to chopped strand on the shielding effectiveness, bandwidth coverage, and reflection behavior within the 0–4.4 GHz range has not been investigated in detail. Filling this gap is essential for the advancement of low-cost, lightweight, and eco-friendly EMI shielding materials for modern high-frequency technologies.

Although kaolinite-containing composites have been explored for microwave-related applications, their material design objectives and composite architectures differ significantly from the present study. Hu et al. [44] developed kaolin-reinforced carbon black/glass fiber/polyamide 6 composites mainly for microwave absorption enhancement in a thermoplastic matrix. In contrast, the present study focuses on microwave shielding effectiveness, evaluated in transmission mode using S-parameter measurements. The proposed epoxy-based kaolinite/chopped-strand composites are designed as thin (1.4 mm) broadband shielding materials operating in the 0.4.4 GHz frequency range, achieving high attenuation without the incorporation of conductive carbon additives or magnetic fillers.

Despite the growing demand for lightweight, low-cost, and environmentally responsible electromagnetic interference shielding materials, most reported polymer-based shielding composites rely on electrically conductive carbon materials, metallic fillers, or magnetic phases. These materials can provide high attenuation but may increase cost, density, processing complexity, and susceptibility to oxidation or interfacial incompatibility. The importance of the present study lies in evaluating a less-explored combination of thermally treated kaolinite, chopped glass strands, and epoxy for microwave attenuation without intentionally incorporating conductive carbon or metallic fillers. This approach uses an abundant mineral filler and industrial glass strands to develop a thin and potentially economical composite system. In addition, investigating the influence of the kaolinite-to-chopped-strand ratio provides useful information on how composition affects transmission, reflection, absorption, and overall shielding effectiveness.

In this work, kaolinite/chopped-strand epoxy composites with different weight ratios were fabricated and systematically investigated. Structural and microstructural properties were analyzed using X-ray diffraction (XRD), scanning electron microscopy (SEM), and energy-dispersive X-ray spectroscopy (EDS). The microwave shielding effectiveness of the composites was evaluated using a vector network analyzer in the 0–4.4 GHz frequency range. The results provide insight into the role of composite composition on shielding performance and demonstrate the potential of kaolinite/chopped-strand epoxy composites as microwave shielding materials.

## 2. Experiment

### 2.1. Raw Materials

Natural kaolinite powder obtained from Balıkesir (Türkiye) was used as the ceramic filler. The kaolinite was supplied as a high-purity mineral raw material (≥99%) with an average particle size of approximately 10 µm and a density of approximately 2.6 g·cm^−3^. Chopped glass fiber strands were commercially supplied by Koloğlu Kimya and consisted of industrial-grade E-glass fibers (≥99% purity) with nominal lengths ranging from 3 to 6 mm.

A commercial CHS 130 epoxy powder, supplied by Spolchemie, Czech Republic, was used as the polymer matrix. The material had a reported viscosity of 0.3–0.6 and an epoxy index of 1.25–1.43. A small amount of hardener powder was added at a mass ratio of 1 g hardener to 24 g epoxy powder. No liquid resin or solvent was used. The powder and mineral/fiber constituents were hot-pressed at 60 °C under 5 MPa for 45 min. Because independent DSC/FTIR confirmation of crosslinking was not performed, the process is referred to as hot pressing or consolidation rather than complete curing. All raw materials were used as received, without further chemical purification [45,46]. The natural kaolinite powder was pressed into pellets approximately 10 mm in diameter and 1–2 mm in thickness using a uniaxial hydraulic press at 2 MPa. The pellets were then calcined at 450 °C for 4 h in a sealed alumina crucible to remove volatile and organic components. After cooling, the calcined material was ground in an agate mortar and used as the kaolinite filler in the composite preparation. No additional sintering or heat treatment was performed.

### 2.2. Preparation of Kaolinite/Chopped Strands Composites

Thermally treated kaolinite-derived powder and chopped strands were mixed in two different weight ratios: 80/20 wt.% and 60/40 wt.% (kaolinite/chopped strands). The chopped glass strands were supplied with a nominal length of 3–6 mm. They were mechanically crushed using a ceramic mortar and subsequently ball-milled in ethanol for 20 h using 10 mm zirconia balls. Because the fiber-length distribution was not measured after milling, the 3–6 mm value should be regarded only as the initial supplier specification and not as the final fiber length in the composites. The resulting slurries were dried at 50 °C for 24 h, filtered, and further dried under vacuum at 50 °C for an additional 24 h. The prolonged milling treatment is expected to reduce the initial strand length and aspect ratio. Accordingly, the electromagnetic discussion has been revised to avoid attributing the shielding response directly to the nominal initial fiber aspect ratio. The chopped glass strands had a nominal initial length of 3–6 mm according to the supplier. However, mechanical crushing followed by 20 h of ball milling was expected to reduce the strand length and aspect ratio. Because the fiber-length distribution was not measured after milling, the 3–6 mm value is considered only the initial supplier specification and not the final fiber length within the composites.

### 2.3. Preparation of Epoxy–(Kaolinite/Chopped Strand) Composites

A commercial bisphenol-A–based epoxy resin powder (DGEBA type) was used as the polymer matrix material. The epoxy resin was employed in solid powder form and consolidated together with the kaolinite and chopped glass strands by hot pressing.

The prepared kaolinite/chopped-strand powders were incorporated into the epoxy system at a constant powder-to-epoxy weight ratio of 4:1 (Table 1). The mixtures were consolidated in a hydraulic hot press at 5 MPa and 60 °C for 45 min. The term ‘consolidated’ is used because the degree of epoxy crosslinking was not independently determined by DSC or FTIR, followed by cooling to room temperature under pressure. The same consolidation conditions and resin formulation were used for all samples to minimize variations in the dielectric properties of the matrix. Accordingly, differences in shielding performance are discussed in terms of the combined interactions among the filler constituents, the epoxy matrix, and the filler–matrix interfaces. All samples were prepared with a nominal thickness of 1.4 mm to determine the performance in terms of microwave shielding properties and were produced in 1.4 mm in thickness considering some feasibility issues concerning the mechanical strength and reliable use in positioning in the co-axial method setup (in a transmission line cell), and based on trial test of different thicknesses. Thinner materials often posed an issue on mechanical stability and hence led to unreliable shielding result, whereas 1.4mm was selected. Although specimen thickness can influence shielding effectiveness, a systematic thickness-dependent investigation was not performed in the present study. Therefore, no conclusion regarding the proportional effect of thickness can be drawn from the available data.

For clarity, the processing sequence consisted of three stages: preparation of the filler mixture, incorporation of the filler mixture into the epoxy powder, and hot pressing of the final mixture. The kaolinite-derived powder and chopped strands were first combined according to the selected 80/20 or 60/40 mass ratio. The epoxy powder was then added at a constant filler-to-epoxy mass ratio of 4:1, corresponding to 20 wt.% epoxy in the final composite. The complete mixture was homogenized before being placed in the pressing mold. The mold was then subjected to 5 MPa pressure at 60 °C for 45 min, followed by cooling to room temperature under pressure. The consolidated plates were subsequently removed from the mold and prepared for microwave measurements. 

The density and porosity of the consolidated specimens were not directly measured. Consequently, the effects of compaction, void content, and matrix distribution on the shielding response cannot be separated from the effects of composition.

The kaolinite/chopped-strand ratios refer to the relative proportions within the inorganic filler fraction. The overall specimen composition is reported separately, including kaolinite, chopped strands, and epoxy. All percentages are given on a total-mass basis.

### 2.4. Characterization Techniques

The phase constitution and crystal structure of kaolinite, chopped strands, and kaolinite/chopped-strand composite powders were performed with X-ray diffraction (XRD) with a Bruker D2 Phaser diffractor (Cu-K ray = 1.5406) in a 2 range of 10–70 at a scan rate of 1 min. The crystalline phases were interpreted based on reference data in the standard PDF database, and lattice parameters of kaolinite were refined with FullProf.

The microstructure and the surface morphology of the sample were observed under a JEOL 5910LV scanning electron microscope (SEM). Elemental composition and phase homogeneity were evaluated by energy-dispersive X-ray spectroscopy (EDS) attached to the SEM system, providing semi-quantitative elemental analysis of the kaolinite grains and composite structures.

Prior to sample measurements, the vector network analyzer and coaxial transmission-line fixture were calibrated to remove systematic errors of cables and connectors and to define the measurement reference planes at the fixture interfaces. A two-port calibration procedure (open/short/load/thru at the fixture ports, according to the manufacturer’s standard calibration routine) was performed before each set of measurements. After calibration, an ‘empty-fixture’ (sample-free) measurement was recorded as a baseline. Composite specimens were then inserted and measured under the same conditions. Shielding effectiveness was calculated from the measured transmission coefficient using the ratio of the transmitted power with and without the sample.

The microwave shielding effectiveness (SE) of the epoxy-based kaolinite/chopped-strand composites was measured using a dual-port vector analyzer (VNA) (Rohde & Schwarz (R&S) FSH-K42) over the frequency range of 0–4.4 GHz. Measurements were conducted using a coaxial transmission-line holder specifically designed to maintain a characteristic impedance of 50 Ω throughout the measurement system. The epoxy–kaolinite/chopped-strand composites were machined into solid coaxial-ring specimens to the dimensions of the inner and outer diameter of the coaxial transmission-line holder for the purposes of microwave shield measurements. The thickness of the specimens was 1.4 mm, completely filling the cross-sectional area of the coaxial sample holder and providing a 100% filling ratio so as to lessen air gaps and thus reducing measurement uncertainty. Each specimen was inserted tightly in between the inner and outer conductor so that a tight mechanical contact occurred at both surfaces for the purpose of measurement stability and ensuring that the incident impedance was matched.

Calculation of electromagnetic shielding efficiency for each composite layer, the electromagnetic shielding effectiveness (SE) was determined from its S-parameters, i.e., reflectance coefficient (S) and transmission coefficient (S), which are measured with a vector network analyzer. The total electromagnetic shielding effectiveness (SET) is the ratio, in dB, of initial incoming energy to transmitted energy according to

*SE_T_* = −10*log*_10_(*T*) (1)

where

T = ∣S_21_∣^2^

T = ∣S_21_∣^2^ represents the power transmission coefficient. Therefore,

*SE_T_ =* −20*log*_10_*|S*_21_*|* (2)

The measured quantity 20*log*_10_|*S*_21_| represents the transmission coefficient in decibels and is negative for attenuating specimens. In this manuscript, shielding effectiveness is reported as the corresponding positive attenuation quantity, *SE_T_ =* −20*log*_10_|*S*_21_|.

The reflection coefficient R and absorption coefficient A are given by

R = ∣S_11_∣^2^ (3)

A = 1 − R − T(4)

Therefore, the total shielding effectiveness can be split in two parts: loss on reflection (SER) and loss on absorption (SEA):

*SE_R_* = −10*log*_10_(1 − *R*) (5)

(6) SET=−10log10T1−R

The total shielding response was measured by considering values for reflection, transmission, and absorption determined from the measured S-parameters. However, the heterogeneous and thin nature of these samples means that no attempt was made to isolate the contribution to transmission or absorption from internal scattering and multiple reflections.

The microwave shielding effectiveness measurements were conducted in the 0–4.4 GHz frequency range, which corresponds to the stable and reliable operating bandwidth of the coaxial transmission-line fixture and calibration employed in this study. Within this range, stable measurement conditions and reproducible S-parameter responses were achieved for the composite samples with a thickness of 1.4 mm. Although coaxial measurement techniques can theoretically extend beyond this range, data outside 0–4.4 GHz were not considered due to increased measurement uncertainty associated with fixture limitations. Measurements in other frequency bands were not performed in the present work and will be addressed in future studies using alternative measurement configurations.

## 3. Results and Discussion

### 3.1. XRD Analysis of Kaolinite-Chopped Strands

**Figure 1:**
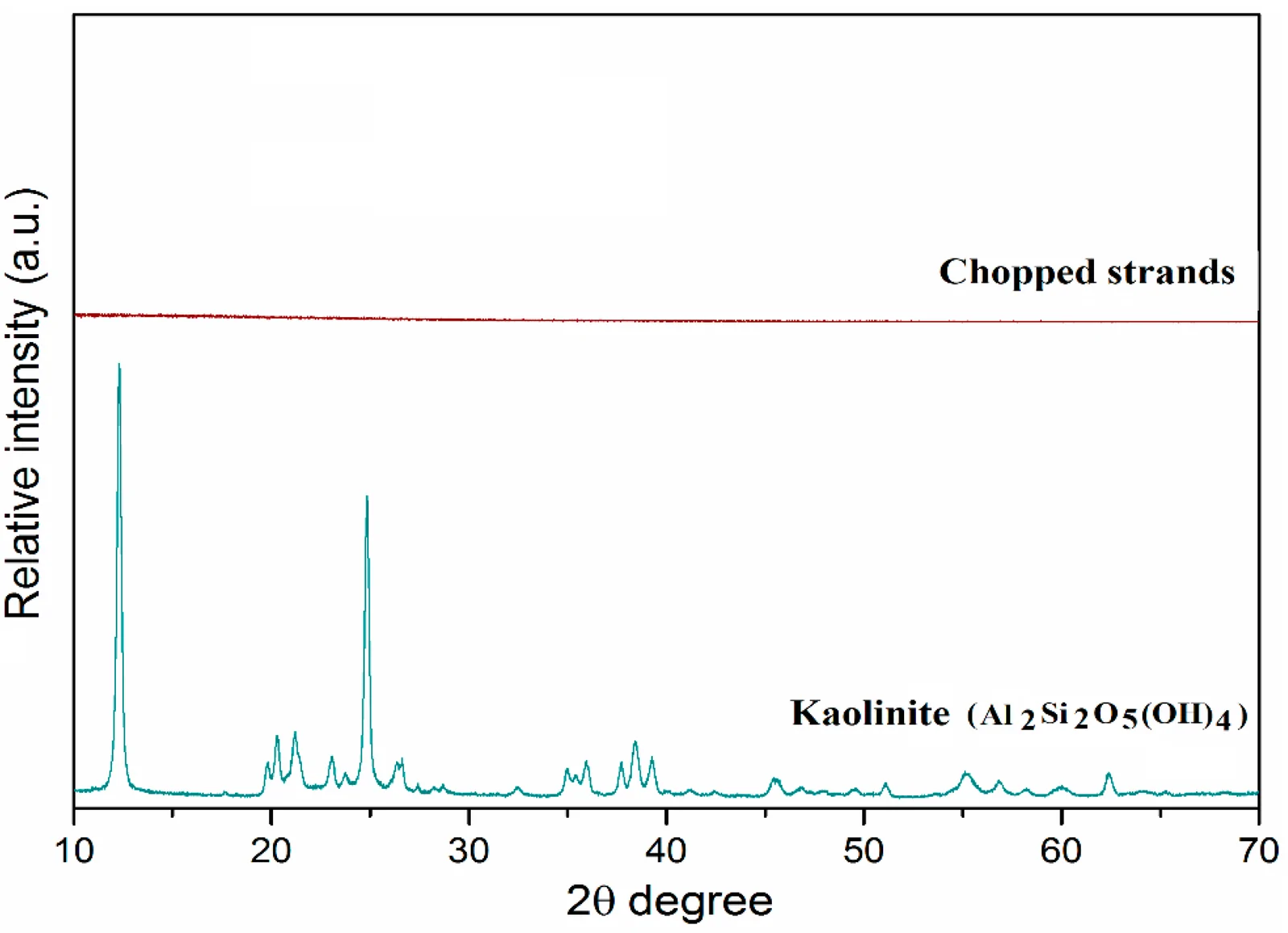
XRD pattern of kaolinite powder after calcination at 450 °C for 4 h.

The crystal structure and phase composition of kaolinite and kaolinite/chopped-strand composites were investigated by X-ray diffraction (XRD), and the corresponding diffraction patterns are presented in Figure 1. The observed reflections are consistent with the kaolinite phase according to PDF card 00-005-0143. Within the detection limits of the measurement, no additional intense crystalline reflections were observed; however, complete phase purity and quantitative phase fractions were not established [41].

The diffraction peaks observed for kaolinite correspond to a triclinic crystal system with space group P1, which is consistent with previously reported structural data [6,41,47]. The refined lattice parameters from the FullProf fitting are listed in Table 2 and the indexed parameters are broadly consistent with reported kaolinite crystallographic data; however, complete Rietveld agreement factors were not determined. Clear peaks which are fairly sharp and intense reflect the high crystallinity of the structure whereas lack of peak splitting/broadening suggests no significant lattice strain [47].

Aside from phase analysis, the peak broadening of X-ray diffraction for the kaolinite phase was used to compute the average crystallite size on the basis of the Scherrer equation:

(7) $$D=\frac{\mathrm{K\lambda}}{\mathrm{\beta cos\theta}}$$

with the shape factor (0.9), the Cu-K wavelength (1.5406Å), the full width at half maximum (FHW) of the diffraction peak in radians, and the Bragg angle, and the estimated average crystallite size was approximately 14–15 nm using the most intensive reflection of the kaolinite. This reflects the coherence size for diffraction, not the size of the larger aggregates and platelets shown in SEM, because kaolinite is a highly layered structure with significant aggregation tendency.

Because XRD patterns of the final composite powders were not obtained, the present data cannot be used to confirm the absence of secondary crystalline phases in the composites.

These values were compared to the unit-cell parameters reported for ideal kaolinite (AlSiO(OH)) from the literature. The parameters of the literature structure were a = 5.1556, b = 8.9397, and c = 7.4073 and the unit cell volume was 329.88.

The corresponding refined parameters in this study, which are a = 5.14, b = 8.93, c = 7.37, and V = 326.99, show minor negative deviations of <0.5% in the lattice constants and ~0.9% in the unit-cell volume. These relatively minor variations fall within the limits of reported deviation of unit-cell parameter from ideal stoichiometry in both natural and processed kaolinite, suggesting an approach toward a structure very near the stoichiometric references. It is, however, important to point out that unit-cell deviations alone are not a reliable measure of chemical non-stoichiometry, because these deviations can be also caused by microstrain effects, preferred orientation, stresses retained following heating treatments, and the inherent incertitude that always remains upon refinement. Therefore, the XRD analysis also confirms the formation of an almost stoichiometric kaolinitic phase with slight structural alterations reflecting processed and microstructural factors.

**Figure 2:**
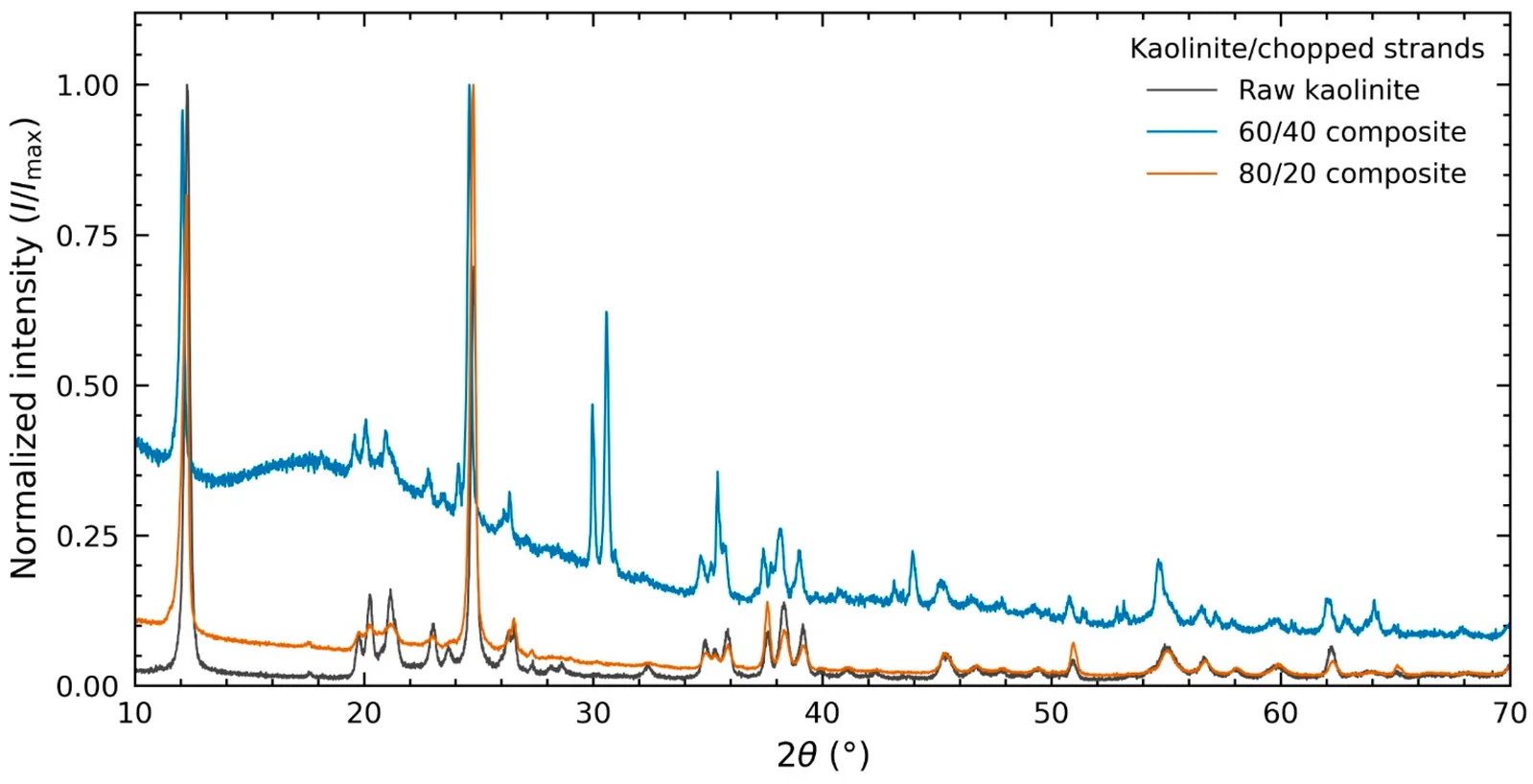
XRD patterns of raw kaolinite and the 60/40 and 80/20 epoxy composites containing kaolinite calcined at 450 °C. The patterns are normalized to the maximum intensity for comparison.

Figure 2 presents the XRD patterns of the raw kaolinite and the kaolinite/chopped-strand epoxy composites. The raw kaolinite pattern exhibits characteristic reflections in the low-angle region, including the strong reflection near 2θ = 12.3 °, together with the reflection near 2θ = 24.9 °, which are associated with the kaolinite structure and are consistent with the reference PDF card 00-005-0143. The presence of these reflections confirms the kaolinite-related crystalline structure of the mineral reference material.

The XRD patterns of both the 60/40 and 80/20 composites retain the principal kaolinite-related reflections, although their relative intensities are reduced and partially overlapped by the broad background contribution from the epoxy matrix. The broad diffuse background observed across the composite patterns is consistent with the contribution of the polymeric phase and indicates the coexistence of crystalline mineral/filler-related regions with the largely amorphous epoxy matrix. The composite patterns do not show an obvious additional crystalline reflection that can be unambiguously assigned to a new secondary phase within the resolution of the measurement.

The differences in peak intensity between the two composite formulations are associated mainly with the different kaolinite-to-chopped-strand ratios and the dilution of the crystalline kaolinite phase by the epoxy matrix and chopped glass strands. The higher kaolinite content in the 80/20 composite produces comparatively stronger kaolinite-related reflections than those observed for the 60/40 composite. However, because the patterns were not subjected to complete quantitative Rietveld refinement and the composite phases were not independently analyzed by complementary techniques, the XRD results should be interpreted as qualitative evidence of phase presence rather than definitive proof of complete phase purity or homogeneous filler dispersion.

The XRD results provide qualitative information about the crystalline phases present in the raw kaolinite and composite specimens; they do not independently confirm the microscale dispersion of the fillers within the epoxy matrix.

### 3.2. SEM Analysis of Kaolinite

**Figure 3:**
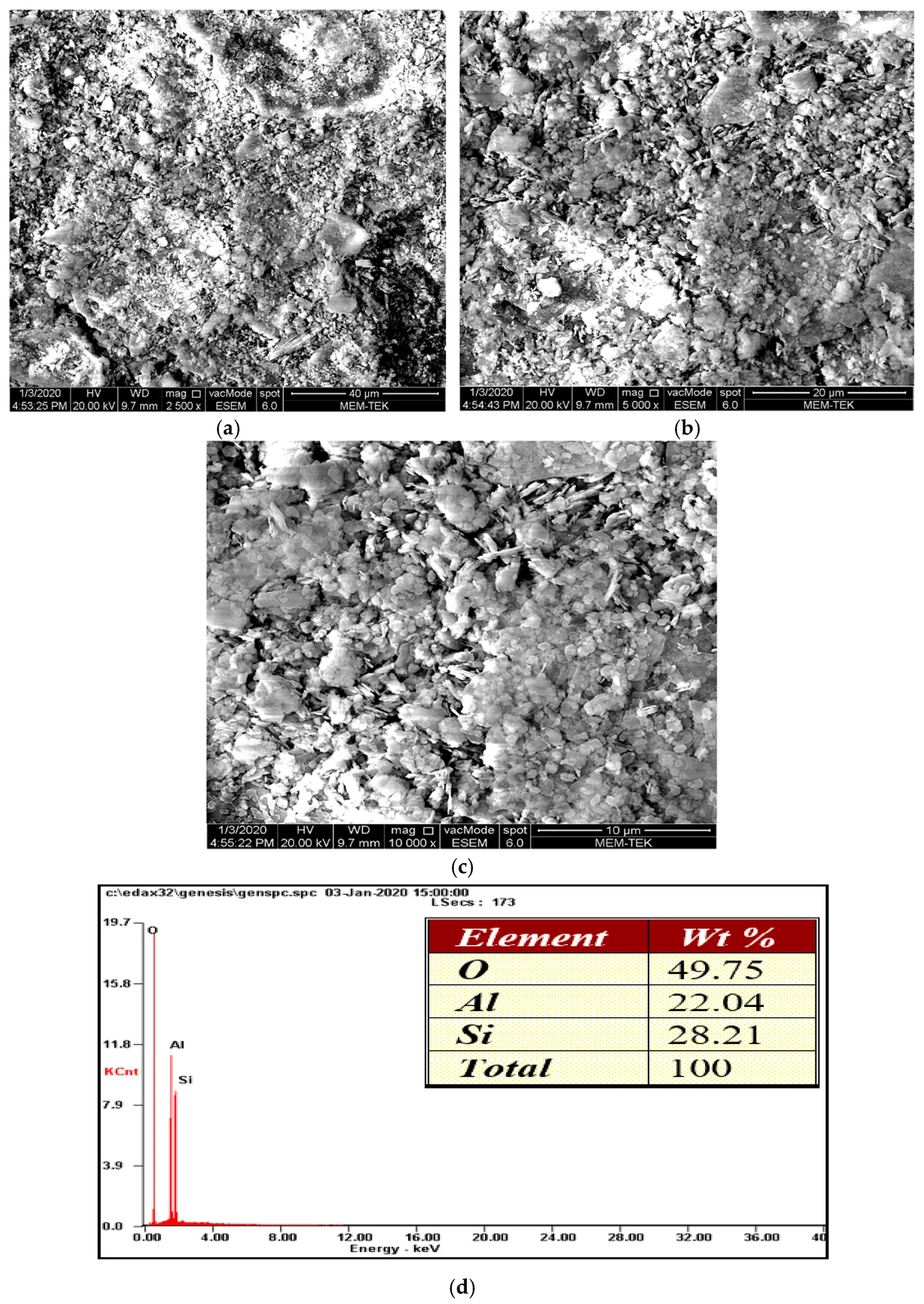
SEM photographs of single-phase kaolinite calcined at 450 °C for 4h (**a**) at ×2500, (**b**) at ×5000, (**c**) at ×10,000, (**d**) EDS analysis of kaolinite at ×5000.

Figure 3 presents the SEM micrograph and EDS spectrum of the kaolinite powder. These data provide information about the morphology and elemental composition of the kaolinite starting material; they do not directly establish the dispersion of kaolinite or chopped glass strands within the epoxy matrix. As depicted in the micrographs, the kaolinite particles in this sample demonstrate morphology of individual lamellar and platelet shape characteristic of layered aluminosilicate minerals. In other words, the particles possess relative consistency in shape and show identifiable platinesses, consistent with the single-phase behavior observed via XRD.

At higher magnification, individual kaolinite particles can be observed as stacks of more closely associated and sometimes coalescing platelets with sharply defined edges.

This layered structure naturally introduces interfaces between individual particles in composite materials and an increased concentration of internal interfaces between the layer stacks within each individual particle. This structure is beneficial for electromagnetic applications because the interfaces serve as active sites that initiate polarization and dielectric relaxation when combined with an appropriate polymer matrix.

The synergistic SEM and XRD structural information complements each other with different length scales, i.e., SEM information describes the morphology and microstructure information of kaolinite at micrometer level, but XRD-based crystallite size describes the nanostructure size of coherent parts among the particle. The multiscale structural information confirms the absorption dominant electromagnetic shielding property in the kaolinite/chopped-strand epoxy composites.

Element composition analyses using energy-dispersive X-ray spectroscopy (EDS) also corroborate the compositional nature of the kaolinite phase, which shows elemental Al:Si:O ratios that match those expected for kaolinite in their respective molar concentrations. As observed in Figure 3, the EDS signal is relatively consistent across the entire observation field, suggesting compositionally homogeneous material and no presence of any spurious phases. Overall, the information obtained from SEM and EDS indicates that the fabrication method used produces composite samples that are physically and structurally homogeneous, while featuring relatively uniform distributions of kaolinite platelets which are essential to realize improved attenuation via multi-scattering and interface polarization mechanisms.

Although the electromagnetic measurements were reproducible, measurement reproducibility alone cannot verify microscale filler dispersion. Therefore, conclusions regarding composite homogeneity are limited by the absence of composite cross-sectional SEM/EDS mapping.

Further studies will incorporate EDS mapping to perform more quantitative evaluation of homogeneity.

Because zirconia balls were used during milling, the possible introduction of trace Zr-containing contamination should be considered. No independent chemical analysis specifically targeting zirconium contamination was performed; therefore, its contribution cannot be completely excluded.

### 3.3. EMI Shielding Effect and Reflection Loss Measurements of Kaolinite/Chopped Strands

**Figure 4:**
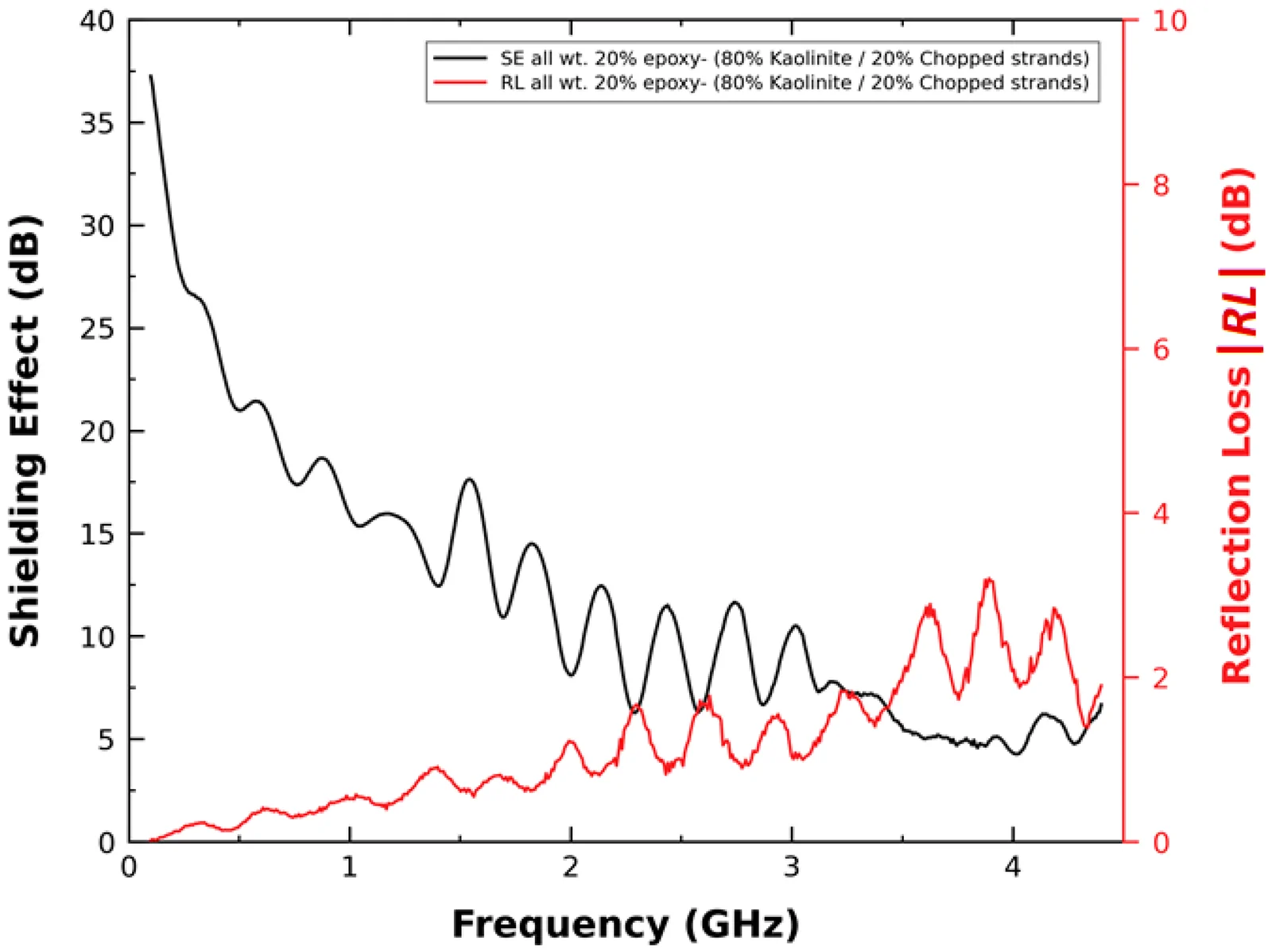
Total shielding effectiveness and Reflection loss of the epoxy–(kaolinite/chopped strands) composites: all wt. 20% epoxy- (80% kaolinite/20% chopped strands) compositions as a function of frequency.

The electromagnetic interference (EMI) shielding effectiveness (SE) and reflection loss (RL) of the epoxy-based kaolinite/chopped-strand composites was systematically evaluated in the 0–4.4 GHz frequency range (Figure 4), and the frequency-dependent SE and RL results are presented in Figure 3 and Figure 4. All measurements were performed on circular specimens with a uniform thickness of 1.4 mm to ensure reliable comparison between different compositions. The shielding effectiveness values presented in this section were calculated directly from the measured S-parameters, ensuring an accurate representation of the transmission and reflection behavior of the composites in the 0–4.4 GHz frequency range.

The results indicate that the shielding effectiveness strongly depends on the kaolinite-to-chopped-strand weight ratio. Among the investigated samples, the composite containing 80 wt.% kaolinite and 20 wt.% chopped strands exhibited the highest shielding performance. This composition achieved a maximum total SE value of 21.57 dB at 0.58 GHz, corresponding to the most attenuation of the incident electromagnetic radiation. Furthermore, this composite yielded shielding effectiveness values below −10 dB in the widest band range of 0 to 1.91 GHz, below 15 dB in the 0 to 1.29 and 1.48 to 1.61 GHz frequencies, and below 20 dB in the 0 to 0.62 GHz frequency band. This composite has not given a reflection loss value below −10 dB.

**Figure 5:**
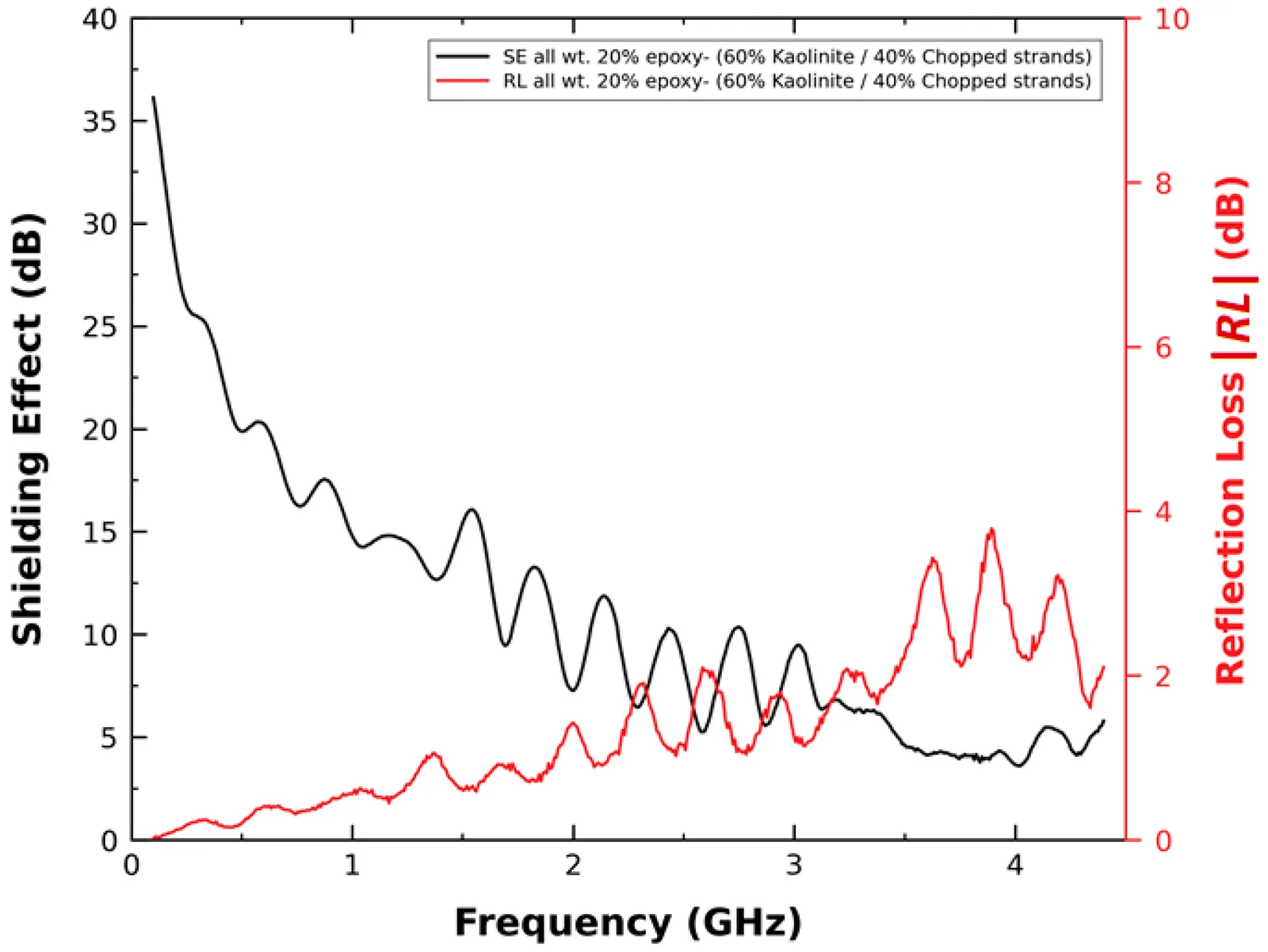
Total shielding effectiveness and reflection loss of the epoxy–(kaolinite/chopped strands) composites: all wt. 20% epoxy- (60% kaolinite/40% chopped strands) compositions as a function of frequency.

In comparison, the composite with a lower kaolinite content (60 wt.% kaolinite and 40 wt.% chopped strands) (Figure 5) showed a slightly reduced peak shielding performance, with a maximum total shielding effectiveness of 20.43 dB at 0.58 GHz. However, this combination yielded SE values below 10 dB and 15 dB in some bands of the 0–4.4 GHz range. In the wide frequency range, it yielded SE values below 10 dB in the 0 to 1.65 GHz range and below 20 dB in the 0 to 0.45 GHz range. The difference in shielding behavior indicates that the kaolinite-to-chopped-strand ratio influences the electromagnetic response; however, increasing the chopped-strand content did not improve the maximum shielding effectiveness under the present processing and measurement conditions. This composite material did not give a reflection loss value below 10 dB; it gave lower reflection loss values compared to the other composite at certain frequencies, for example, it gave 3.44, −3.81, and 3.24 dB RL values at 3.63, 4.09, and 4.19 GHz frequencies, respectively.

This observed shielding effect may be interpreted to be due to a mixture of the absorption-based mechanisms; dielectric loss, interfacial polarization, and scattering within the heterogeneous structure of the composite material. Chopped strands not only introduce yet another set of interfaces, but also alter the electromagnetic behavior of the heterogeneous composite. Within the heterogeneous composite, they contribute to the polarization, scattering, and dissipation of energies within the material. These numerous interfaces (between kaolinite, chopped strand, and epoxy matrix) can serve as good polarization losses, or be scattered and dissipated.

The other interesting finding is that due to the thin thickness (1.4 mm) of the composites, the EMI shielding performance even approaches or surpasses previous EMI shielding materials with higher thicknesses (as presented in Table 3), especially at the frequency lower than 3.0 GHz. This suggests that controlling the filler ratio is promising to develop either broadband EMI or frequency-selective shielding performance. The EMI shielding measurements showed that the kaolinite/chopped-strand epoxy composites present simultaneously high EMI SE, broad bandwidth and small thickness. Thus kaolinite/chopped-strand composites are excellent candidate materials to be used for the EMI shielding in applications of high-frequency communication, radar systems and electromagnetic compatibility.

We must remark that SE spectra of Figure 4 oscillate locally about their average value, displaying local minimum and maximum values. Such oscillations happen in typical coaxial transmission-line measurement settings of the interface at the fixture sample and, being likely ascribed to multiple reflections or standing-wave phenomena at impedance mismatches, may be particularly notable for the relatively thin composite samples. Thus, they are not a direct indication of intrinsic absorption resonant behavior.

Herein, the shielding behavior will be assessed mostly based on the attenuation values and coverage over a broad bandwidth (e.g., keeping SE < 10 dB) consistently reproduced among measurements. In order to comparatively analyze the compositions, shielding behavior was quantitatively described beyond the SE spectra, adopting the following: (i) total bandwidth average SE from 0 to 4.4 GHz and (ii) frequency intervals where the SE threshold values of SE < 10 dB and SE < 20 dB are fulfilled (Table 4).

In this study, a total shielding effectiveness of SE_T_ ≥ 10 dB is considered to represent at least 90% attenuation of the incident electromagnetic power. Values of SET < 10 dB correspond to attenuation below 90%. These thresholds refer to the positive shielding-effectiveness convention and should not be confused with the negative S_21_ values measured by the vector network analyze.

### 3.4. Reflection Behavior and Its Relation to Shielding Performance

The reflection behavior of the epoxy–kaolinite/chopped-strand composites was evaluated using the measured S_11_ parameter. The power reflection coefficient is expressed as R = ∣S_11_∣^2^, while the corresponding reflection-loss values provide an indication of the fraction of incident electromagnetic energy reflected at the sample interface. Because only the magnitude-based reflection data are considered in the present analysis, complex input impedance and its separate real and imaginary components are not reported.

The reflection-loss results show that neither composite exhibit RL values below −10 dB within the investigated 0–4.4 GHz frequency range. For the composite containing 80 wt.% kaolinite and 20 wt.% chopped strands, representative RL minima of 2.89, 3.79, and 2.86 dB were obtained at 3.62, 3.89, and 4.19 GHz, respectively. The composite containing 60 wt.% kaolinite and 40 wt.% chopped strands exhibited RL values of 3.44, 3.81, and 3.24 dB at 3.63, 4.09, and 4.19 GHz, respectively (Table 5).

These results indicate that the strong shielding effectiveness observed in the composites should not be interpreted solely in terms of minimized surface reflection or ideal impedance matching. Instead, the measured S-parameter results are consistent with attenuation arising from the combined contributions of reflection, transmission reduction, and absorption within the heterogeneous composite structure. In particular, the high shielding effectiveness observed at lower frequencies occurs without correspondingly large negative RL values.

Therefore, the shielding performance of the present composites is more appropriately evaluated using the measured transmission- and reflection-derived shielding parameters rather than a complex impedance-matching analysis. Further determination of the frequency-dependent complex input impedance would require phase-resolved complex S-parameter measurements.

## 4. Discussion

Rather than relying solely on local minima or maxima in frequency-dependent curves, the correlation between composite composition and shielding performance is evaluated using band-integrated metrics derived from Table 6. These results demonstrate that increasing the chopped-strand content from 20 wt.% to 40 wt.% did not increase the maximum shielding effectiveness. Instead, the maximum total shielding effectiveness decreased from 21.57 dB for the 80/20 composite to 20.43 dB for the 60/40 composite, while the two compositions showed comparable broadband coverage within the investigated frequency range. This quantitative comparison supports a composition–performance correlation within the scope of the investigated ratios.

The shielding effectiveness (SE) for microwave absorption of the kaolinite/chopped-strand epoxy composites is influenced significantly by the composite composition, the microstructural features, and the interfacial interactions among the constituent components. These results show that considerable effective shielding properties for EMI can be achieved using the lightweight and thin layers for the composite (1.4 mm). A consistent dependence of the shielding effectiveness on the ratio of the chopped strands to kaolinite has been noticed.

For the composite filled with 80 wt.% of kaolinite and 20 wt.% of chopped strands, a maximum value of shielding effectiveness was up to 21.57 dB at 0.58 GHz with other several intervals of SE under 10 dB in the measuring frequency range of 0–4.4 GHz. For the composite containing 60 wt.% kaolinite and 40 wt.% chopped strands, the maximum total shielding effectiveness was 20.43 dB at 0.58 GHz. Although this value was lower than that of the 80/20 composite, the 60/40 formulation maintained a measurable shielding response over a wide frequency range.

The observed attenuation is consistent with an absorption-dominated response based on the measured reflection, transmission, and absorption characteristics derived from the S-parameters. However, the specific physical mechanisms responsible for this behavior were not independently verified by dielectric spectroscopy or complex-permittivity measurements. Dielectric loss and interfacial charge accumulation may contribute to the attenuation because of the dielectric heterogeneity among the kaolinite-derived powder, chopped glass strands, and epoxy matrix.

The interfaces among the kaolinite-derived particles, chopped strands, and epoxy matrix may act as sites for charge accumulation and interfacial polarization, potentially contributing to dielectric loss. However, this interpretation cannot be quantitatively confirmed without direct dielectric-property measurements.

The plate shape and high density of the internal surfaces in kaolinite leads to the increase in number of inside scattering center as the reason to obtain the EM wave multiple reflection. Increasing the path of EM wave propagation will lead to increasing the total absorbed magnitude. An interfacial polarization created between kaolinite together with the chopped strands and the epoxy matrix as the improvement of the interface density needed for attenuated effect can happen.

Measurements showed the fluctuation characteristic according to frequencies; sharper and peak-shaped shielding effect could be due to the resonator behavior caused by geometry of compound, filler arrangement, and holder of measurement which is very typical to this kind of heterogeneous shielded system with significant electromagnetic coupling of incident waves with sample, and the spreading of the shielding peaks means that more than one relaxation polarization process exists, leading to broadband shielded behavior. Additional effects that are responsible for high loss are again multiple reflection effects, being more pronounced in thin samples. Reflections between interfaces can amplify or detract from shielding effectiveness by considering the impedances matching with absorption power. In the present composites, since a porous sample was used, together with the shredded fibers, by the process of kaolinite combined with chopped fiber, a process that could decrease the multiple reflection’s influence was dominated, that is, absorbing dominated shielding effect.

The present composite system is obviously different from previously reported microwave materials composed of kaolinite in design principle and function orientation. Different from the focus on microwave absorption in terms of reflection-loss properties in previous reports, we are more interested in microwave shielding ability in terms of transmission loss. A high shielding effectiveness for small thickness depends on synergistic contributions of kaolinite microstructure, polarization of the interfaces between kaolinite and chopped strands/epoxy resin, as well as the loss mechanism related to heterogeneous morphology inside the composites.

In this respect, we highlight a mineral-based filler and glass fiber system with an effective broadband shielding achieved without inclusion of conductive or magnetic fillers.

The kaolinite/chopped-strand composites obtain comparable or enhanced shielding effectiveness over the reported values (Table 3) at a similar thickness but avoid using costly conductive fillers and magnetic fillers. In addition to abundant kaolinites and chopped strands being commercially available, easy preparation process and tunable performance also contribute to the practical interest. Given the identical composition of polymer matrix, mixing ratio and curing process are used in all the cases investigated, varying shielding properties can be well attributed to difference in the composition as well as in the ensuing heterogeneity and the consequent interfacial structure.

The reflection-loss data further demonstrated that high shielding effectiveness was realized without tremendously large negative RL. Thus, the shielding mechanism should not be simply attributed to the low surface reflection and it is more relevant to be analyzed with reflection, transmission, and absorption features extracted from measured S-parameters. In summary, effective microwave shielding can be realized based on such co-synergistic effect between kaolinite and chopped strands which mainly consists of loss associated with the interfacial polarization loss, dielectric loss, and multiple scattering within heterogeneous composite. Further control over filler ratios provides efficient control over shielding effectiveness and working bandwidth for broadband EMIs which can be applicable to radar systems, wireless communication, and EMI compatibility related areas.

### Microwave Shielding Mechanism of Kaolinite/Chopped-Strand Epoxy Composites

It is important to separate reflection loss (RL), as frequently encountered in studies of microwave absorbers, from absorption-related shielding effectiveness (SEA) as formulated by Schelkunoff. Unlike reflection loss that measures how well impedance-matched surface (under typically metal-backing condition) is performing in the air region, SEA deals with what energy is absorbed within the electromagnetic shielding bulk. As the SEA determined from measurement takes into the volume of a shield, absorption performance should be judged with the SEA, not the conventional RL of the absorber. In this work, therefore, the criterion of using the S-parameter-derived SEA for the Schelkunoff model is applied to assess the microwave absorption shielding performance.

In this work, the microwave shielding response appears to include a substantial absorption-related contribution based on the calculated reflection, transmission, and absorption parameters. The response may involve dielectric loss, interfacial polarization, and multiple scattering within the heterogeneous composite structure. Because the complex dielectric properties were not measured independently, these mechanisms should be regarded as possible explanations rather than experimentally confirmed processes.

Dielectric loss mainly can occur from dipole polarization. First, the kaolinite particles in the layered structure of aluminosilicate can contribute strongly dipolar polarization. Due to the intrinsic defects present as a result of its processing and preparation techniques, it also contains charged-related polarizations which lead to dielectric relaxation loss under alternating fields in the way that the lagging of polarization after field variation will result in the loss. In addition to that, there is inherent dipole polarization of the epoxy matrix. It interacts strongly with ceramic filler.

Interfacial polarization may arise from the contrast in electrical and dielectric properties among the kaolinite-derived powder, glass strands, and epoxy matrix. Such heterogeneity can promote charge accumulation at phase boundaries and may contribute to Maxwell–Wagner–Sillars polarization. Nevertheless, complex permittivity, dielectric-loss tangent, and impedance data were not independently measured in this study. Therefore, the contribution of Maxwell–Wagner–Sillars polarization remains a plausible interpretation rather than a directly verified mechanism [44].

The proposed attenuation mechanisms should be interpreted as composite-level effects rather than as independently verified contributions from each constituent. In particular, the present experimental design did not include neat epoxy, epoxy/kaolinite, or epoxy/chopped-strand control specimens. Consequently, the separate effects of the polymer matrix, thermally treated kaolinite-derived powder, and chopped glass strands cannot be quantified directly. The observed response is therefore attributed to the combined influence of the heterogeneous composite structure, interfacial polarization, dielectric losses, multiple scattering, and specimen morphology.

Once electromagnetic waves enter the material, scattering and internal reflections can occur at filler–matrix interfaces, increasing the effective propagation path and thereby promoting energy dissipation within the composite. The relatively low magnitude of the measured RL values, together with the substantial transmission attenuation, indicates that the observed shielding behavior cannot be explained solely by strong surface reflection [51].

Although detailed dielectric relaxation and complex impedance analyses were not performed in the present study, the observed broadband shielding behavior and S-parameter-derived reflection, transmission, and absorption characteristics are consistent with contributions from dielectric loss and interfacial polarization in the heterogeneous composite system.

## 5. Limitations of the Present Study

Several limitations should be acknowledged. Composite-level SEM/EDS mapping and direct density/porosity measurements were not performed in the present study. Consequently, the relationship between microscale filler dispersion, porosity, and electromagnetic attenuation cannot be established conclusively. The proposed mechanisms involving interfacial polarization and multiple scattering should therefore be regarded as plausible interpretations rather than direct experimental proof. Future work should combine cross-sectional SEM/EDS mapping, density, and porosity analysis, and control specimens to clarify these relationships.

The post-milling fiber-length and aspect-ratio distributions were not measured. In addition, possible zirconia contamination from the milling media was not independently quantified.

Several processing-related limitations should be acknowledged. The degree of epoxy crosslinking and the glass-transition temperature were not independently determined by DSC or FTIR. In addition, the fiber-length and aspect-ratio distributions after milling were not measured, and possible zirconia contamination from the milling media was not quantified. Systematic thickness trials and direct density/porosity measurements were also not performed. Consequently, the effects of matrix conversion, post-milling fiber morphology, residual porosity, and specimen thickness on the electromagnetic response cannot be isolated conclusively. These parameters should be investigated in future work.

Control specimens consisting of neat epoxy, epoxy/kaolinite, and epoxy/chopped strands were not evaluated. Therefore, the individual contributions of the epoxy matrix, kaolinite-derived powder, and chopped glass strands to the measured electromagnetic response cannot be separated conclusively. The differences between the 80/20 and 60/40 composites represent the combined effect of composition, filler distribution, interfacial interactions, porosity, and specimen morphology. Future work should include these control formulations under identical processing and measurement conditions to quantify the contribution of each constituent.

The final fiber-length and aspect-ratio distributions after mechanical crushing and 20 h of ball milling were not measured; therefore, their specific influence on filler dispersion and electromagnetic behavior could not be quantified.

The roles of dielectric loss and Maxwell–Wagner–Sillars interfacial polarization remain interpretative because complex permittivity, dielectric-loss tangent, and complex impedance were not measured independently. The measured S-parameters demonstrate the electromagnetic attenuation response, but they do not by themselves quantitatively separate the contributions of dielectric loss, interfacial polarization, multiple scattering, and other possible mechanisms. Future work should include broadband dielectric spectroscopy and complex-permittivity measurements to verify and quantify these contributions.

Regarding thermal-treatment and phase-analysis limitations, the kaolinite used in this study was calcined at 450 °C for 4 h, and no 1150 °C sintering step was performed. Therefore, the present study does not include XRD data for a 1150 °C-treated powder or a direct analysis of phase evolution up to that temperature. TG-DSC analysis, complete Rietveld refinement statistics, and detailed temperature-dependent XRD measurements were not performed. Consequently, the degree of dehydroxylation and the detailed thermal evolution of the kaolinite cannot be established conclusively. The possible contribution of hydroxyl-related polarization is therefore considered tentative.

The composite XRD patterns provide qualitative evidence of the retained kaolinite-related reflections and the contribution of the epoxy matrix. However, complete Rietveld refinement, quantitative phase analysis, and complementary thermal analysis were not performed. Therefore, the XRD results cannot conclusively establish the exact phase fractions or fully exclude minor secondary phases.

## 6. Conclusions

In this study, epoxy-based kaolinite/chopped-strand composites were successfully fabricated and systematically investigated for their electromagnetic interference (EMI) shielding effectiveness in the 0–4.4 GHz frequency range. Composites with different kaolinite-to-chopped-strand weight ratios were prepared, and their structural, microstructural, and electromagnetic properties were evaluated to assess their suitability for microwave shielding applications.

X-ray diffraction and SEM/EDS analyses were used primarily to characterize the kaolinite powder and the principal features of the composite constituents. Because composite-level SEM/EDS mapping and quantitative phase analysis were not performed, definitive conclusions regarding filler homogeneity, phase purity, and the absence of secondary phases cannot be drawn. The lamellar morphology of kaolinite and the effective interfacial contact between kaolinite particles, chopped strands, and the polymer matrix contributed to the formation of multiple internal interfaces, which are favorable for electromagnetic wave attenuation. Results for microwave shielding confirmed that the composite composition significantly influences shielding performance. For composites with 80 wt% kaolinite and 20 wt% chopped strands, shielding performance was very effective as maximum shielding effectiveness was measured as 21.57 dB at 0.58 GHz, while the values measured in some of the bands of the frequency range between 0 and 4.4 GHz were below −10 dB at 1.4 mm thickness. At low kaolinite concentration, the composite with 60 wt% kaolinite and 40 wt% chopped strand is seen to be also showing a good and stable shielding performance in wide frequency range, however, with a smaller highest value of shielding.

It was found that efficient EMI shielding characteristics of composite are due to the combined behavior of different loss mechanisms, namely, dielectric loss, interface polarization, as well as the multiple scattering and internal reflection behaviors within composite materials’ heterogeneous architecture. With these parameters, the kaolinite-chopped-strand composite ratio could become useful for designing low-cost and environmentally friendly composite to improve their shielding performance across the broadband and frequency dependent characteristics.

This work shows the potential applications of the kaolinite/chopped-strand epoxy composites as environmentally friendly, cost effective, and lightweight EMI shielding material in high-frequency ranges. Furthermore, research is useful in the way of modifying its composite design and its use in other ranges of frequency.

## Figures and Tables

**Table 1:** The overall compositions of the raw materials.

Sample	Kaolinite	Chopped Strands	Epoxy
80/20 filler ratio	64 wt.%	16 wt.%	20 wt.%
60/40 filler ratio	48 wt.%	32 wt.%	20 wt.%

**Table 2:** Using FullProf, computed lattice parameters of a kaolinite sample.

Sample	a (Å)	b (Å)	c (Å)	Volume of Cell (Å^3^)	Space Group	SYS
Kaolinite	5.14	8.93	7.37	326.99	P1 (1)	Anorthic (Triclinic)

**Table 3:** Comparison of reported electromagnetic-shielding parameters for the present and previously reported materials.

Sample	Thick (mm)	SE (dB)	Freq. (GHz)	Ref.
All wt. 20% epoxy- (80% Kaolinite/20% chopped strands)	1.4	21.57	0.58	This work
Wollastonite/PANI/Colemanite	1.5	41.65	6.26	[37]
CF/epoxy composite	2	29.4	12.4	[38]
BaFe_12_O_19_ nanoparticles	3	17	11.58	[39]
Graphene nanocomposite	22.86 × 10.16 × 2	30	X band	[40]
rGO/AgNW/PET	0.192	27	8.2–12.4	[48]
Ta doped NiFe_2_O_4_	1.2	34.74	17.4	[49]
NiFe_2_O_4_/rGO	2	38.2	10.8	[50]

Note: The reported electromagnetic values were obtained under different material compositions, specimen thicknesses, frequency ranges, and measurement configurations. The reported parameter is identified as total shielding effectiveness (SE_T_, reflection loss (RL), absorption shielding effectiveness SE_A_, or transmission coefficient S_21_, according to the original source. Direct comparison should therefore be made cautiously.

**Table 4:** Shielding effect of the epoxy–(kaolinite/chopped strands) composites with the ranges of shielding characteristics in GHz.

Sample	SE (dB)	Frequency (GHz)
All wt. 20% epoxy- (80% kaolinite/20% chopped strands)	21.57	0.58
	18.82	0.88
	16.06	1.16
	17.77	1.54
	14.63	1.82
	12.54	2.14
	10	0–1.91
		2.07–2.19
		2.39–2.48
		2.69–2.79
	15	0–1.29
	15	1.48–1.61
	20	0–0.62
All wt. 20% epoxy- (60% kaolinite/40% chopped strands)	20.43	0.58
	17.58	0.88
	16.25	1.54
	13.4	1.82
	10	0–1.65
		1.74–1.89
		2.09–2.18
	15	0–0.96
		1.49–1.55
	20	0–0.45

**Table 5:** Reflection loss of the epoxy–(kaolinite/chopped strands) composites with the ranges of shielding characteristics in GHz.

Sample	RL (dB)	Frequency (GHz)
All wt. 20% epoxy- (80% kaolinite/20% chopped strands)	−2.89	3.62
	−3.79	3.89
	−2.86	4.19
All wt. 20% epoxy- (60% kaolinite/40% chopped strands)	−3.44	3.63
	−3.81	4.09
	−3.24	4.19

**Table 6:** Quantitative correlation between composite composition and shielding performance derived from Table 4.

Composition (Kaolinite/Chopped Strands)	Max SE (dB)	Frequency Range with SE ≤ 10 dB (GHz)	Total Bandwidth with SE ≤ 20 dB (GHz)	Fraction of Band with SE ≤ 10 dB
80/20	21.57 @ 0.58 GHz	0–1.91	≈0.62	≈50.45%
60/40	20.43 @ 0.58 GHz	0–1.65	≈0.45	≈42.95%

## Data Availability

Data will be available upon request.
